# A nutraceutical diet based on *Lespedeza spp*., *Vaccinium macrocarpon* and *Taraxacum officinale* improves spontaneous feline chronic kidney disease

**DOI:** 10.14814/phy2.13737

**Published:** 2018-06-14

**Authors:** Alessandro Di Cerbo, Tommaso Iannitti, Gianandrea Guidetti, Sara Centenaro, Sergio Canello, Raffaella Cocco

**Affiliations:** ^1^ Department of Life Sciences University of Modena and Reggio Emilia Modena Italy; ^2^ Department of Medical, Oral and Biotechnological Sciences Dental School University G. d' Annunzio of Chieti‐Pescara Chieti Italy; ^3^ KWS BioTest Portishead Somerset UK; ^4^ Research and Development Department SANYpet S.p.a Padua Italy; ^5^ Research and Development Department Forza10 USA Corp Orlando Florida; ^6^ Department of Pathology and Veterinary Clinic Faculty of Veterinary Medicine University of Sassari Sassari Italy

**Keywords:** Aspartate aminotransferase, blood urea nitrogen, cat, chronic kidney disease, creatinine, feline, total proteins, urine color score, urine turbidity score

## Abstract

Chronic kidney disease is characterized by structural and/or functional impairment of one or both kidneys persisting for more than 3 months. In cats, chronic kidney disease can frequently occur in animals aged over 9 years with an incidence of approximately 10%. Thirty‐four client‐owned, neutered cats, suffering from stage II‐III chronic kidney disease and diagnosed according to the International Renal Interest Society guidelines were randomly assigned to receive either a control diet (*n* = 17) or a nutraceutical diet (ND;* n* = 17) for 90 days. Both diets were commercialized for management of CKD symptoms. The diets were identical except that the ND contained tablets that consisted of 60–80% hydrolysed proteins, 20–40% minerals and active substances, that are, *Lespedeza* spp. 0.0588%, *Vaccinium macrocarpom* 0.0371%, and *Taraxacum officinale* 0.0231%. No adverse effects were reported during this study. Both diets resulted in an improvement in CKD symptoms. After a 90‐day evaluation, creatinine, blood urea nitrogen, total proteins, and aspartate aminotransferase significantly decreased in cats that received the ND. A significant decrease was also observed in urine turbidity score, color score, and total proteins in cats that received the ND. We have found that a ND based on *Lespedeza spp*., *Vaccinium macrocarpon*, and *Taraxacum officinale* improves key indicators of renal failure in cats affected by chronic kidney disease.

## Introduction

Chronic kidney disease (CKD) is defined as structural and/or functional impairment of one or both kidneys persisting for more than 3 months (Shanan et al. 2017). CKD is irreversible and ultimately progressive. In cats, CKD can occur at all ages being more frequent in animals aged over 9 years with an incidence of approximately 10% (Finch et al. 2016). Clinically, CKD results in body weight loss, decrease in muscle mass, and an unkempt appearance. Polyuria and polydipsia occur because of the kidney inability to regulate water balance (Bartges 2012). Hyporexia, anorexia, vomiting, halitosis, ulcerative stomatitis, and gastroenteritis may also occur. The diagnosis of CKD is obtained by palpating the kidneys that appear small and irregular in the presence of CKD. In addition, abdominal radiography and ultrasonography are also used to aid diagnosis. Biochemically, azotemia with inappropriately dilute urine, metabolic acidosis, and hyperphosphatemia are present (Fortney 2012). Additionally, some animals may have hypokalemia (seen more commonly in cats than in dogs), non‐regenerative anemia, hypoalbuminemia, dyslipidemia, and urinary tract infection. Proteinuria may also occur and has been associated with a poor prognosis and more rapid progression of CKD (Cannon 2016). The kidneys play a key role in homeostasis through filtration, reabsorption, and secretion (Raila and Schweigert 2001). After the diagnosis of CKD has been made, staging is achieved by evaluating serum creatinine concentrations on two occasions when the patient is well hydrated, urine protein‐to‐serum creatinine ratio on two or three occasions, and indirect arterial blood pressure assessment on two or three occasions (Agarwal 2003; Bartges 2012; Bian and Shang 2011). CKD is staged depending on the magnitude of renal dysfunction and the presence or absence of proteinuria and/or hypertension (Buoncompagni and Bowles 2014; Vaden and Elliott 2016). Although early diagnosis of CKD is considered an important factor for positive prognosis, serum creatinine concentration and urine specific gravity assessment at 1–2 years of age and at the beginning of each year from 5 to 10 years of age should be considered to ensure a better quality and a longer life span (Grauer 2005; Sparkes et al. 2016). To date, the only pharmacological approach for CKD treatment is the management of its symptoms, for example, nausea and anorexia (Ljutic et al. 2002; Quimby et al. 2011) and the attempt to reduce intraglomerular hypertension (Agarwal 2003). Thus, dietary intervention becomes of key importance in CKD treatment in cats to restore key blood and urine physiological parameters.

In this study, we tested the hypothesis that a dietary intervention based on the combination of nutraceutical substances, a low content of phosphorus, potassium, calcium and proteins derived from extensive farming would improve CKD in cats.

## Material and Methods

### Animals and study design

Thirty‐four client‐owned, neutered cats [23 European, 3 Persian, 3 British short hair and 5 Chartreux cats [13,59 ± 0.86 years (mean age ± standard error of the mean) and 4.71 ± 0.26 Kg (mean weight ± standard error of the mean); 65% females and 35% males], suffering from stage II–III chronic kidney disease diagnosed according to the International Renal Interest Society guidelines (Cannon 2016) and manifesting at least one symptom among vomit, polyuria, polydipsia, halitosis, oral ulcer, weight and appetite loss, lethargy, depression, dehydration, and weakness, were enrolled in this randomized controlled clinical evaluation performed at the University of Sassari, Department of Veterinary Medicine, Pathology and Veterinary Clinic Section (Sassari, Italy).

Immediately following the diagnosis of CKD, the cats were randomly assigned to receive either a control diet (CD; *n* = 17) or a nutraceutical diet (ND; *n* = 17) for 90 days according to the manufacturer's instructions ensuring that an equal number of cats with CKD stage II and stage III was included in each treatment group (Table 1). The cats were enrolled at different times within a 30‐day timeframe and then evaluated for 90 days.

**Table 1:** Suggested daily amount of nutraceutical diet to be administered to each cat based on its body weight according to the manufacturer's instructions

Weight (Kg)	Amount of diet (g)
1–3	20–40
3–4	40–50
4–6	50–70
6–8	70–85

Operative procedures and animal care were performed in compliance with national and international regulations (Italian regulation D.L.vo 116/1992 and EU regulation 86/609/EC). The CONsolidated Standards Of Reporting Trials (CONSORT) 2010 guidelines were also followed (Bian and Shang 2011). Moreover, the International Society of Feline Medicine consensus guidelines for the management of cats with CKD were followed (Sparkes et al. 2016).

### Diets

Both diets were commercially available for management of CKD in cats and shared similar analytical constituents and additive contents in compliance with the nutrient requirements of dogs and cats of the National Research Council of the National Academies (Council 2006). Both CD and ND contained dry kibbles (~93–94% in weight), a low content of phosphorus, sodium, proteins, potassium, and calcium but the ND also contained cold‐pressed tablets at the 6–7% in weight of complete food (European patent *n*. 2526781). The tablets consisted of 60–80% hydrolysed proteins (fish and vegetable proteins) and 20–40% minerals used as glidants, and were enriched with active substances (*Lespedeza* spp. 0.0588%, *Vaccinium macrocarpom* 0.0371%, and *Taraxacum officinale* 0.0231%).

### Biochemical urine analysis

All cats received veterinary inspections at baseline and 30, 60, and 90 days. Urine and hematological analyses were performed at baseline (T0) and at the end of the evaluation (T1). Dipstick urinalysis was performed using Multistix^®^ 10 SG^®^ reagent strips (Siemens S.p.A, Milan, Italy). The reagent strip contained test pads for pH, urine specific gravity, and proteins. Urine color was graded according to the following score: dark = 0, clear = 1, and very clear = 2. Turbidity was graded according to the following score: limpid = 0, partially turbid = 1, and turbid = 2 (Canello et al. 2017). Hematological analyses [creatinine, blood urea nitrogen (BUN), phosphorus, potassium, calcium, albumin, hematocrit, alanine aminotransferase (ALT), and aspartate aminotransferase (AST)] were performed using Dimension RxL Max Integrated Chemistry System (Siemens Healthcare Diagnostics S.r.l., Milan, Italy).

### Statistical analysis

Data were analyzed using GraphPad Prism 7 software (GraphPad Software, Inc., La Jolla, CA). All data are presented as the means ± standard error of the mean and were first checked for normality using the D'Agostino‐Pearson normality test. All the data were normally distributed and the difference in hematological and urinary parameters between the two groups was analyzed using a two‐way analysis of variance (ANOVA) followed by Sidak's *post hoc* test for multiple comparisons.

## Results

**Figure 1:**
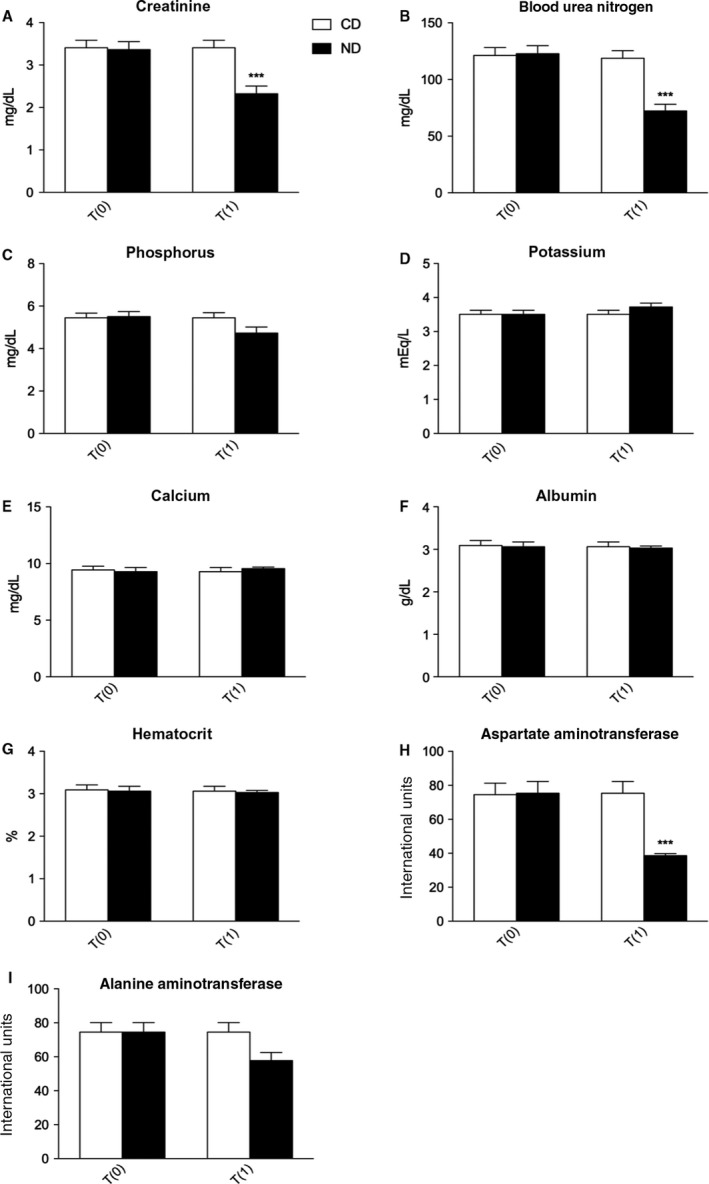
Changes in hemato‐biochemical parameters in cats affected by CKD and treated with control diet (CD) or nutraceutical diet (ND) before (T0) and after 90 days (T1) of diet administration. ***P < 0.001

Diets were administered according to bodyweight, as described in Table 1, with an average of ~50 g of diet daily. The cat owners reported that each cat always consumed their daily allowance. Both diets resulted in an improvement in CKD symptoms, that is, vomit, polyuria, polydipsia, halitosis, oral ulcer, weight and appetite loss, lethargy, depression, dehydration, and weakness, as determined by the owners in the form of observations. No adverse effects were reported during this study. After 90 days of evaluation, creatinine, BUN, and AST significantly decreased in cats that received the ND (Fig. 1). In particular, creatinine decreased from a baseline value of 3.36 ± 0.18 to 2.32 ± 0.17 mg/dL (*P *<* *0.001), BUN decreased from a baseline value of 122.8 ± 6.95 to 72.29 ± 5.94 mg/dL (*P *<* *0.001), and AST decreased from a baseline value of 75.41 ± 6.90 to 38.65 ± 1.18 international units (I.U.) (*P *<* *0.001). No significant changes in hematocrit, calcium, phosphorus, potassium, albumin and ALT were observed (Fig. 1).

**Figure 2:**
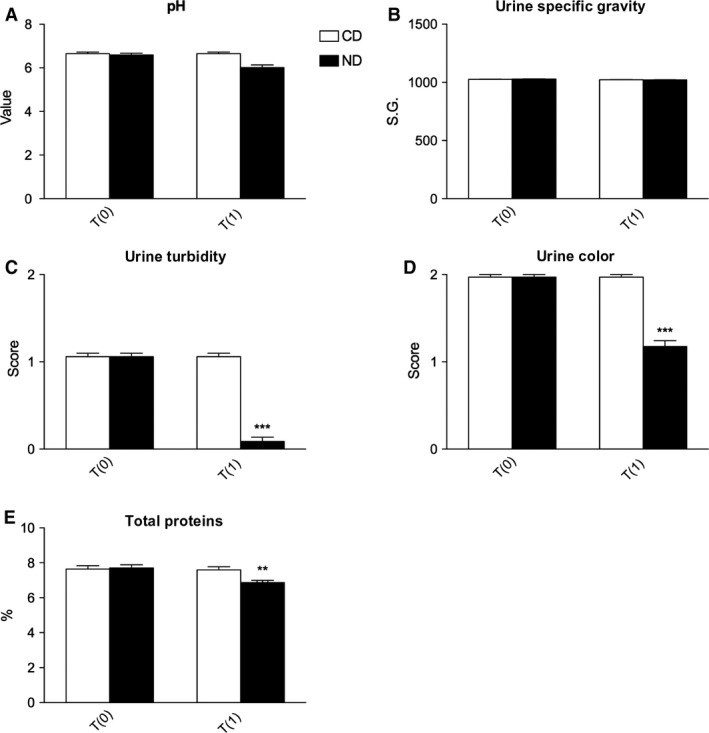
Changes in urine parameters in cats affected by CKD and treated with control diet (CD) or nutraceutical diet (ND) before (T0) and after 90 days (T1) of diet administration. **P < 0.01 versus CD and ***P < 0.001 versus CD

As to urine parameters, a significant decrease was observed in urine turbidity score, color score, and total proteins in cats that received the ND (Fig. 2). Urine turbidity score decreased from a baseline of 1.05 ± 0.04 to 0.08 ± 0.04 (*P *<* *0.001). Urine color score decreased from a baseline of 1.97 ± 0.02 to 1.17 ± 0.06 (*P *<* *0.001). Total proteins decreased from a baseline of 7.70 ± 0.18% to 6.87 ± 0.12% (*P *<* *0.01). No significant changes in pH, and urine specific gravity were observed (Fig. 2).

## Discussion

We have found that ND, a diet based on *Lespedeza capitata*,* Vaccinium macrocarpon*, and *Taraxacum officinale* improves serum creatinine, BUN, AST, urine color score, and urinary protein concentration in cats affected by CKD. ND administration resulted in an improvement in CKD symptoms similarly to the CD, which, however, did not improve hemato‐biochemical parameters. Limitations of this study are (1) the lack of a group of healthy cats receiving the ND to confirm that the changes in hemato‐biochemical parameters are directly caused by the diet itself and (2) the fact that, since food consumption was reported by the cat owners, we cannot rule out the possibility that the changes in biochemical parameters that we observed in this study could simply be related to changes in the dietary intake beyond the diet object of this investigation.

Research conducted on multiple species has repeatedly confirmed the high content of flavonoids and related compounds in *Lespedeza capitata* (Calo et al. 1969; Yarnell 2012). For instance, proanthocyanidins from *Lespedeza capitata* inhibit angiotensin‐converting enzyme in vitro thus exerting an anti‐hypertensive effect (Wagner and Elbl 1992). In addition, polyphenols and flavonoids from *Vaccinium macrocarpon* (cranberry) reduce blood pressure through a reduction in ornithine decarboxylase activity and inhibition of cyclooxygenases (McKay and Blumberg 2007; Ruel and Couillard 2007; Perez‐Lopez et al. 2009). As a matter of fact, hypertension is a consequence of a low glomerular filtration rate, which typically characterizes renal failure (Thomas and Thomas 2009). Moreover, in vivo studies have revealed increased plasma superoxide dismutase activity and reduced nitrate, nitrite, and malondialdehyde (MDA) concentrations in castrated rats after a 4‐month supplementation with cranberry juice (Deyhim et al. 2007). As to *Taraxacum officinale,* a decrease in serum alcaline phosphatase, gamma glutamyl transferase, and glutathione‐S‐transferase enzyme activities and MDA and glutathione levels was observed in the rat kidney (Karakus et al. 2017).

Concentration of serum creatinine, a breakdown product of muscle creatine phosphate, increases progressively as glomerular filtration rate (GFR) declines and is therefore used as a clinical marker of kidney function (Lalor et al. 2009; Scherk and Laflamme 2016). Therefore, an improvement in creatinine levels following ND administration suggests an increase in GFR, which indicates an improvement in renal dysfunction. BUN is a metabolite linked to protein tissue turnover and is also an indicator of renal function (Hosten 1990). In this study, ND improved BUN, which paralleled the effect seen on creatinine. A similar trend was observed for total proteins and urine color score that were also decreased following ND administration (Geddes et al. 2016).

A previous study showed that AST serum levels were correlated with creatinine concentration in patients with pre‐dialysis CKD (Fabrizi et al. 2001). A further study showed that AST and ALT serum levels tended to be higher during the initial stages (2 and 3) of CKD compared with the later stages (4 and 5) (Sette and Lopes 2015). Similarly, in this study, ND significantly decreased AST in cats affected by stage 2 and 3 CKD. This decrease paralleled with the significant decrease in creatinine that was observed following ND supplementation.

CKD can also be worsened by several compounds, for example, nonsteroidal anti‐inflammatory drugs, glucocorticoids, immunosuppressant drugs, and antibiotics (Bartges 2012). For example, we previously reported the presence of an antibiotic, oxytetracycline (OTC), widely employed in intensive farming (particularly chicken and turkey), that binds to the calcium present in animals' bone (Odore et al. 2015; Di Cerbo et al. 2016; Mazzeranghi et al. 2017). It is noteworthy that bone meal and poultry by‐products encompass a 20–30% of commercial pet diets, thus representing an important source of OTC (Di Cerbo et al. 2018). OTC residues can accumulate within pets' body possibly leading to kidney injury. In light of these observations, it is conceivable to speculate that a chronic intake of contaminated food (e.g. OTC‐containing food) can induce a chronic inflammatory status, which can foster or exacerbate kidney injury. We suggest that a diet, based on *Lespedeza capitata*,* Vaccinium macrocarpon*, and *Taraxacum officinale* that improves serum creatinine, BUN, AST, urine color score and urinary protein concentration in cats, could also be employed to counteract drug‐induced worsening of chronic kidney disease. This warrants further investigations.

## Conflict of Interest

This research was not funded by any grant and performed in collaboration with scientists from the Division of Research and Development, Sanypet S.p.a. (Padova, Italy) and Forza10 USA Corporation (Orlando, USA) (GG, SCE and SCA).
